# Morphometrics of the Spinal Cord and Surrounding Structures in *Alligator mississippiensis*

**DOI:** 10.3390/biology11040514

**Published:** 2022-03-27

**Authors:** Skye Greer, Michael J. Cramberg, Bruce A. Young

**Affiliations:** Department of Anatomy, Kirksville College of Osteopathic Medicine, Kirksville, MO 63501, USA; sa204910@atsu.edu (S.G.); mcramberg@atsu.edu (M.J.C.)

**Keywords:** meninges, archosaurs, Crocodylia, vertebrae, cerebrospinal fluid

## Abstract

**Simple Summary:**

Morphometric analysis of the spinal cord and surrounding tissue of the American alligator (*Alligator mississippiensis*) reveals that there are four significantly discrete regions; cervical, thoracic, lumbar, and caudal. Crocodylians, unlike mammals, have a caudal spinal cord that extends throughout the length of their tail (which accounts for roughly 50% of their total body length). *Alligator mississippiensis* has one of the largest ranges of body sizes among terrestrial vertebrates, this study documents how the different spinal structures change with increasing body size. Though most of the structures exhibit slightly positive allometry, a few exhibit slightly negative allometry; these differences mean that there are significant relational changes as hatchlings grow into large adults. This study provides the first documentation that *A. mississippiensis* has an expansive subdural space, a lumbar cistern, at the pelvis.

**Abstract:**

Understanding the fluid dynamics of the cerebrospinal fluid requires a quantitative description of the spaces in which it flows, including the spinal cord and surrounding meninges. The morphometrics of the spinal cord and surrounding tissues were studied in specimens of the American alligator (*Alligator mississippiensis*) ranging from hatchlings through adults. Within any size class of alligators (i.e., hatchlings), along the axial length there are significant differences in the size of the spinal cord, meninges, and vertebral canal; these differences can be used to define discrete cervical, thoracic, lumbar and caudal regions. When compared across the range of body sizes in *Alligator*, every structure in each spinal region had a distinctive growth rate; thus, the physical arrangements between the structures changed as the alligator grew. The combination of regional differentiation and differential growth rates was particularly apparent in the lumbar meninges where a unique form of lumbar cistern could be identified and shown to decrease in relative size as the alligator ages. This analysis of the spinal cord and surrounding tissues was undertaken to develop a data set that could be used for computational flow dynamics of the crocodilian cerebrospinal fluid, and also to assist in the analysis of fossil archosaurs.

## 1. Introduction

The cerebrospinal fluid (CSF) nourishes and supports the central nervous system, but many aspects of the dynamics of the CSF remain poorly known [1]. The challenges associated with gathering invasive experimental data on the CSF, coupled with increasing sophistication of available software, has promoted a growing emphasis on quantitative modelling of CSF dynamics. Most commonly, this is done under the rubric of computational fluid dynamics (CFD), an established approach that is used to explore a variety of biological fluid systems [2,3]. As [4] noted in their recent review, “anatomically accurate descriptions of the CSF spaces...are needed to specify realistic CFD model domains and set boundary conditions.” In human studies, this typically involves using MRI data from a limited patient pool to quantify and model one region of the spinal cord [5]. This approach has yielded some remarkable findings, both for the unexpected flow characteristics of the CSF [6] and for the functional relationship between CSF flow dynamics and spinal pathologies [7,8].

Despite these impressive advances, there are still some basic aspects of human spinal CSF dynamics that are poorly known. The ventilatory cycle appears to be the primary agent of spinal CSF flow [9,10], but the underlying mechanics are not clear. Though generally assumed to be caused by intrathoracic pressure altering central venous pressure, and thus CSF pressure [11,12]; experimental studies have reported significant spinal CSF pressure cycles after great vessel ligation and even exsanguination [13], and the same ventilatory CSF pulses occur in non-diaphragmatic vertebrates [14]. The complete picture of the sources and sinks of human CSF is still developing [15,16], but there is strong evidence from animal studies that there is significant spinal CSF loss along the spinal nerves, via subarachnoid granulations and/or associated lymphatic vessels [17,18]. At the same time, it is currently unknown how (or if) the rate of CSF loss at the spinal nerves varies, and if in humans there is a gradient in loss rates at C2 versus L2 [19]. The human lumbar cistern functions as a CSF fluid reservoir, capable of expanding to contain CSF displaced by cranial trauma [20]. Infusion studies have revealed aspects of the potential dynamic range of the lumbar cistern [21]; less is known about the typical volumes of this space, and how it relates to rostral and caudal spinal CSF flow [22,23]. Nothing is known about how this reservoir function is accomplished in non-mammalian vertebrates.

The understanding of spinal CSF dynamics in non-mammalian vertebrates is far more limited. As previously noted [24], there is considerable morphological variation in the vertebrate spinal cord; particularly in the relationship between the spinal cord and the vertebral canal. This variation, coupled with variation in the presence and size of the lumbar cistern [25], and the different ventilatory mechanics [26,27], suggest that the dynamics of spinal CSF flow may be fundamentally different in some non-mammalian vertebrates.

In a classic paper in vertebrate paleontology, Giffin [28] analyzed the morphometrics of the trunk vertebrae and vertebral canal of *Alligator mississippiensis*, and compared the results to other archosaurs (dinosaurs and birds). Giffin limited her study [28] to the trunk, which makes up only roughly 50% of the alligator’s (or other crocodylian) vertebral column [29,30,31]; the present study was designed to cover the entire spinal cord.

**Figure 1:**
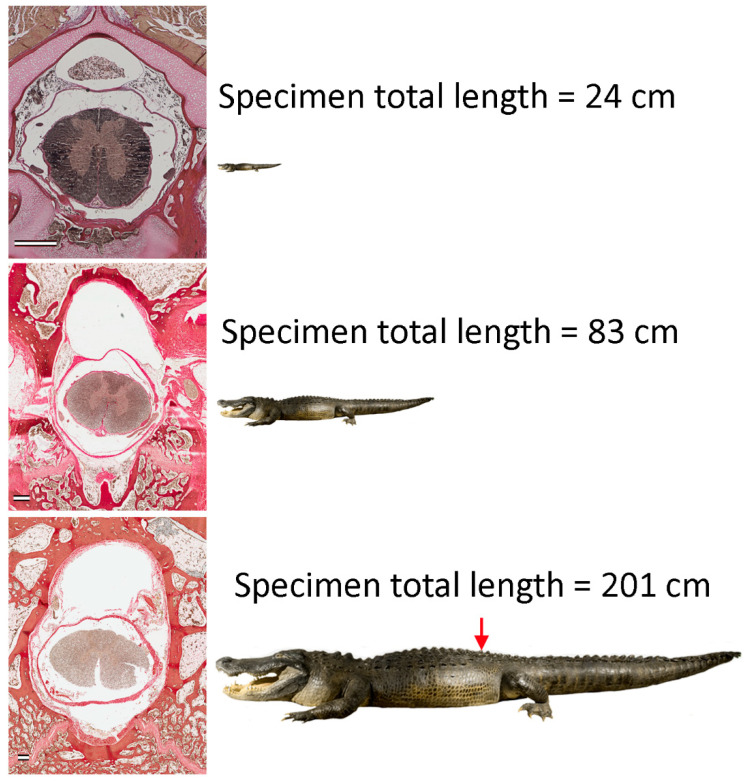
The growth of *Alligator mississippiensis* changes the physical relationships between the spinal cord and adjacent tissues. By comparing sections through the same level of the vertebral column (red arrow), across the range of body sizes, differential growth of the morphological features can be documented. The figures of the alligator have been adjusted to reflect the actual size relationships among the three specimens. To show the three sections at the same size, they have been subjected to different enlargements as reflected in the scale bars of each figure, which are all equal to 1 mm.

The goal of this study was to document changes in the dimensions of the vertebral canal, spinal cord, and dura both along the axial length of the alligator, and as the alligator increased in size. Most morphometric studies of the spinal cord, whether focused on human or other vertebrate taxa, have been restricted to a single size class [32,33,34]. *Alligator mississippiensis* has one of the largest size ranges among terrestrial vertebrates [35]; sampling over this size range could provide a rare perspective on allometric growth of the spinal cord [36] in a non-mammalian vertebrate (Figure 1). The results of this project could form a natural extension of Giffin’s study [28]. Additionally, the resulting quantitative data on the spinal cord and vertebral canal could provide the requisite anatomical data set for a CFD model of the CSF dynamics in *Alligator mississippiensis*.

## 2. Materials and Methods

### 2.1. Specimens

Sixteen *Alligator mississippiensis* were used in this study, ranging in size from hatchlings to adults. Most of these animals came from the Louisiana Department of Wildlife and Fisheries, others were obtained commercially. The majority of the animals were housed in captivity for 6–12 months while being used in unrelated scientific studies. These captive specimens were euthanized through initial anesthesia with isoflurane, followed by cardiac excision and exsanguination. The *Alligator* euthanasia protocol used in this study, as well as all captive maintenance of the animals, was approved by the Institutional Animal Care and Use Committee of the Kirksville College of Osteopathic Medicine (Protocol #175, approved 8/2016).

### 2.2. Samples

**Figure 2:**
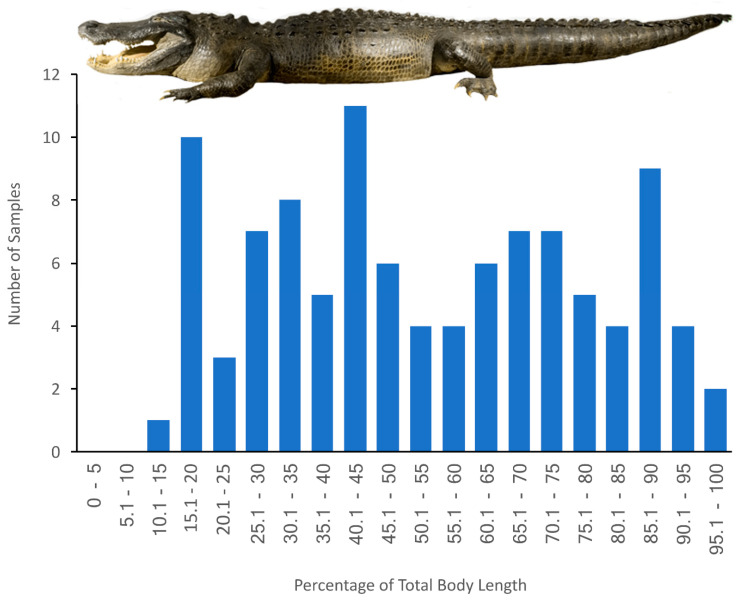
Total samples examined along the body of *Alligator mississippiensis.* No sections were taken from the skull (which accounts for roughly 16% of the total length of the alligator) or from the pelvis (which is located near 50% but shifts slightly during ontogeny). Multiple samples were taken from each individual alligator; samples taken from the same individual were always separated by at least 10% of total body length.

A minimum of six samples were taken from each specimen, typically three pre-sacral specimens and three caudal specimens. In six specimens, additional caudal samples were taken (Figure 2). All samples were complete transverse sections through the vertebral column, including the spinal cord and meninges. In the hatchlings, each sample was only 2–3 mm in thickness; in the larger specimens, the samples could exceed 1 cm in thickness. All samples were removed immediately after the animal was euthanized. In the hatchlings, some samples could be removed with a razor blade. In larger specimens, a combination of bone and reciprocal saws were employed; best results were obtained using a portable band saw (Bauer 1678E-B). Digital photographs were taken immediately after the samples were removed.

### 2.3. Anatomical Preparation

Each sample was fixed in neutral-buffered formalin (nbf) at 4 °C for a minimum of 72 h. Post-fixation the blocks were trimmed of scalation, then decalcified (RDO Rapid Decalcifier) for 48 h. Following this initial decalcification, some of the smaller samples were ready for histological processing; the majority of the samples were trimmed with razor blades then decalcified for an additional 48 h prior to histological processing. Samples were trimmed to facilitate sectioning, while retaining the complete vertebral canal. Samples were dehydrated through an ethanol series, cleared in Xylene, then embedded in Paraplast. Transverse sections were cut (at 10 μm); enough sections were cut from each sample so that multiple slides were produced that contained complete transverse sections through the spinal cord, dura, and the bony margin of the vertebral canal. Sections were stained with a Van Gieson’s/Weigert’s Iron Hematoxylin stain [37]. The histological sections were photographed using a DM 4000B microscope (Leica Microsystems Inc., Tokyo, Japan).

### 2.4. Quantification

**Figure 3:**
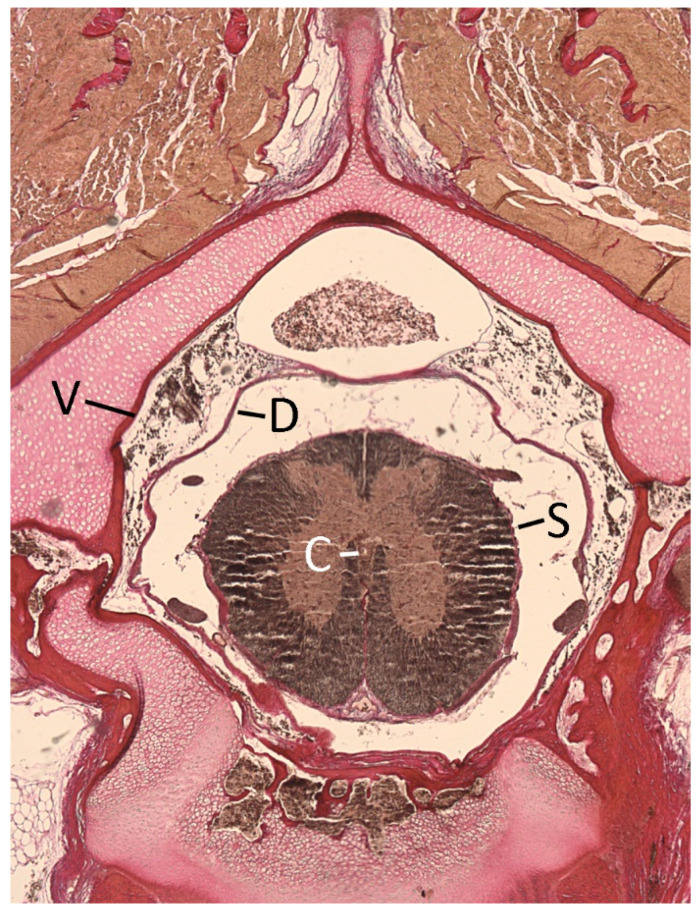
Transverse section through the cervical region of a hatchling specimen of *Alligator mississippiensis*. C—central canal; D—dura; S—spinal cord; V—inner margin of the vertebral canal.

Digital images of each sample were imported into ImageJ [38] with which the actual contours of anatomical structures/spaces could be quantified (rather than trying to fit geometric shapes). Four areas were quantified: central canal of the spinal cord, outer surface of the spinal cord, dura mater, and inner surface of the vertebral canal (Figure 3). Each measurement was repeated five times. If the standard error of the five measurements exceeded 3% of the mean, the five values were erased and the measurements repeated. If the standard error of the five measurements was less than 3% of the mean, the mean value of the five measurements was used for subsequent analyses (see below). To minimize any sampling bias, all of the variables were quantified from the same histological section. This section was chosen from among the slides (typically at least 20) from each vertebral sampling point due to the section’s clarity and lack of artifact or obvious distortion.

The same measurements were taken on a subset of the gross sample images that were taken prior to fixation or histological processing. Comparing the gross image to the histological section from the same sample, provided a test of repeatability and a metric for the magnitude of shrinkage/distortion caused by the histological preparation [39,40].

### 2.5. Statistical Analysis

**Figure 4:**
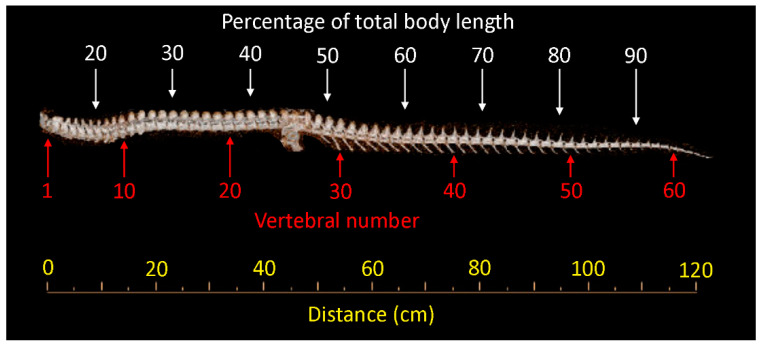
The complete vertebral column of a 148 cm specimen of *Alligator mississippiensis*. The image is based on a 3-D reconstruction of a CT analysis. The vertebral column is labelled with vertebral number and distance (below) and percentage of total body length (above). Note that the percentage of total body length includes the skull (not shown).

The goal of this study was to document changes in the dimensions of the vertebral canal, spinal cord, and dura both along the axial length of the alligator, and as the alligator increased in size. Giffin [28] clearly demonstrated the presence of a cervical, thoracic, and lumbar (our terminology) region in the alligator vertebral column. Ideally, all of these measurements would be referenced against either vertebral or spinal nerve number. Both the small size of the hatchling tail and collecting data in the wild from freshly culled animals prevented us from using this approach. For repeatability, applicability to every size specimen used in this study, and clear correlation with vertebral number (Figure 4), we are using percent of total body length as our indicator of position along the axial length of the alligator.

**Figure 5:**
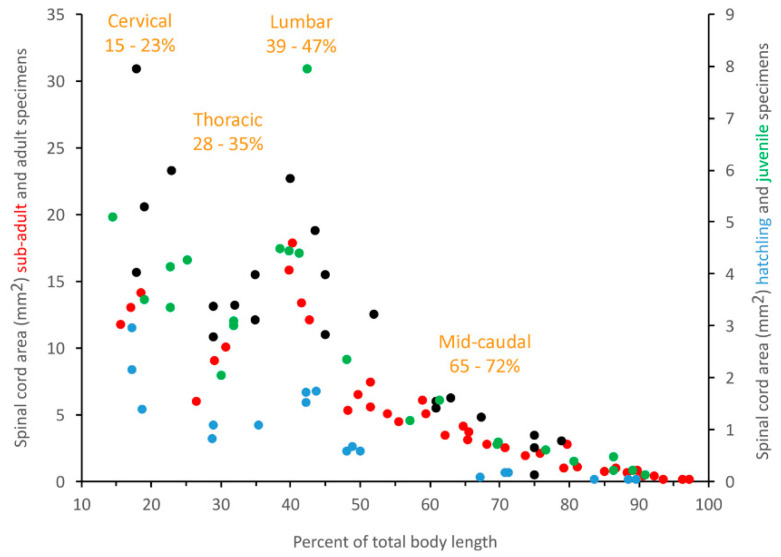
The discrete regions within the spinal cord and vertebral column. The boundaries between these segments are based on significant differences in morphological features. Here, spinal cord area (in mm^2^) is plotted along the length of the vertebral column in hatchling (blue), juvenile (green), sub-adult (red), and adult (black) specimens. Note that the hatchling and juvenile specimens are plotted on the right *y*-axis, while the sub-adult and adult specimens are plotted on the left *y*-axis. In all four size groups the differences in spinal cord areas allow for the definition of a cervical, thoracic, and lumbar regions (in consistent ranges of percent total body length). For comparison, we are defining a portion of the caudal sequence (65–72% of total body length) as a mid-caudal region.

**Figure 6:**
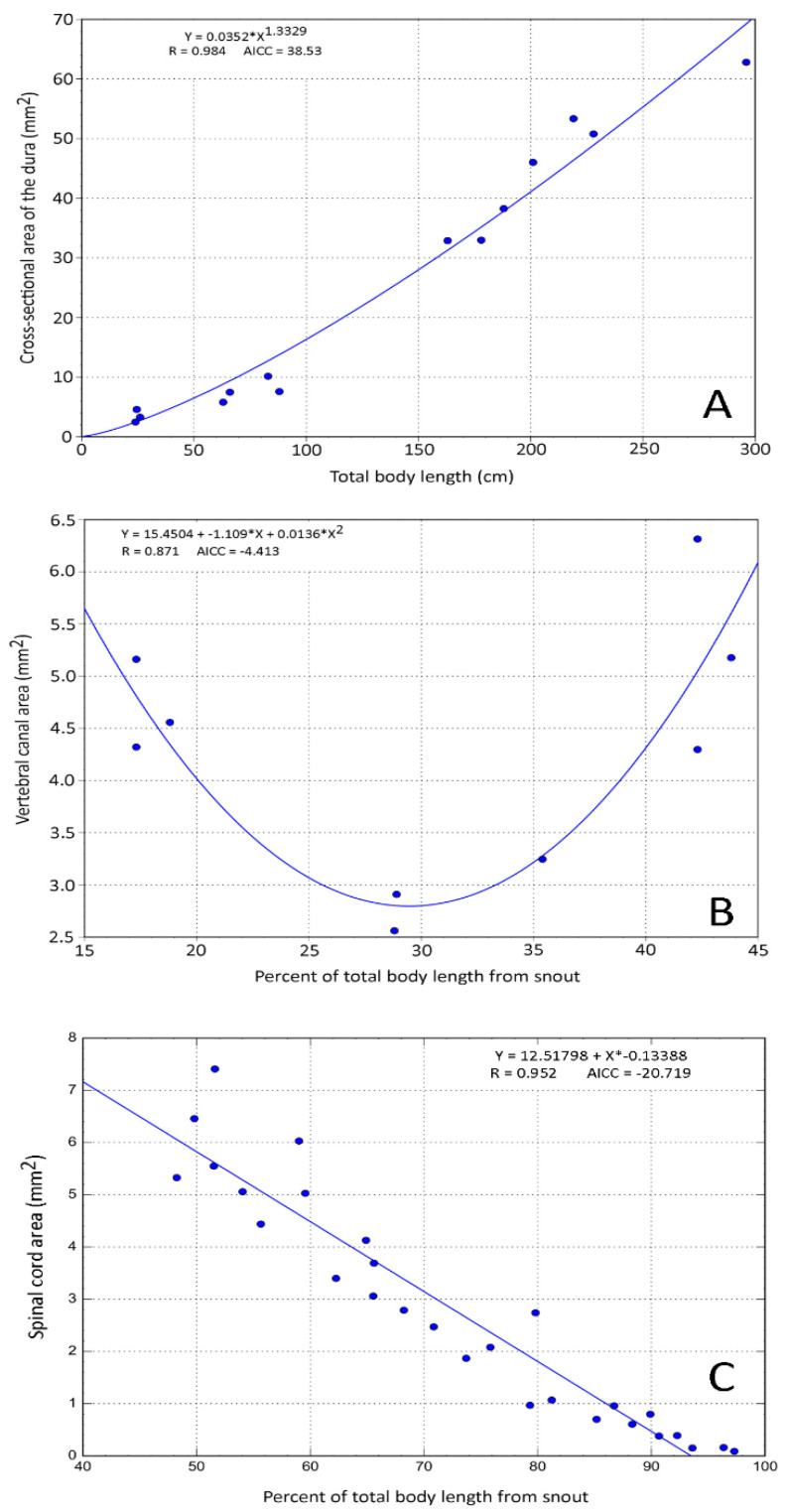
Different curve fitting techniques used on the morphometric data. (**A**) Power curve, used to explore the size of a feature over total body length; (**B**) second-order polynomial curve, used to depict the differences between the cervical, thoracic, and lumbar regions; and (**C**) linear regression, used to depict changes in size of the features along the length of the tail.

Plotting any of our morphometric data (except the area of the central canal, see below) against percent of total body length (Figure 5) revealed a rather consistent pattern that we used to define the cervical (15–23% of total body length), thoracic (28–35% of total body length), lumbar (39–47% of total body length), and mid-caudal (65–72% of total body length) regions. To examine relative growth rates, the ImageJ data from each of these four body regions were pooled, then regressed against total body length using CurveExpert Professional (Hyams Development). For consistency with previous crocodilian studies [41], and to maximize curve fit, we performed non-linear regression analysis (Figure 6A) using the Power model (Y = a ∗ X^b^).

To examine differences between different locations along the axial length of the alligator, we pooled the specimens into four groups: hatchlings (N = 3, total body lengths 24–26 cm), juveniles (N = 4, total body lengths 63–88 cm), sub-adults (N = 5, total body lengths 143–188 cm), and adults (N = 4, total body lengths 201–296 cm). The pooled data sets were compared using MANOVA, with a post hoc Bonferroni test; in both tests, 0.001 was used as the *p* value threshold for significance. The pooled trunk data were fit using second order polynomial equations (Y = a + b ∗ X + C ∗ X^2^; Figure 6B), while the pooled caudal data were fit using linear regression (Y = a + b ∗ X; Figure 6C).

## 3. Results

### 3.1. General Appearance and Variation in the Alligator Spinal Cord and Vertebral Canal

**Figure 7:**
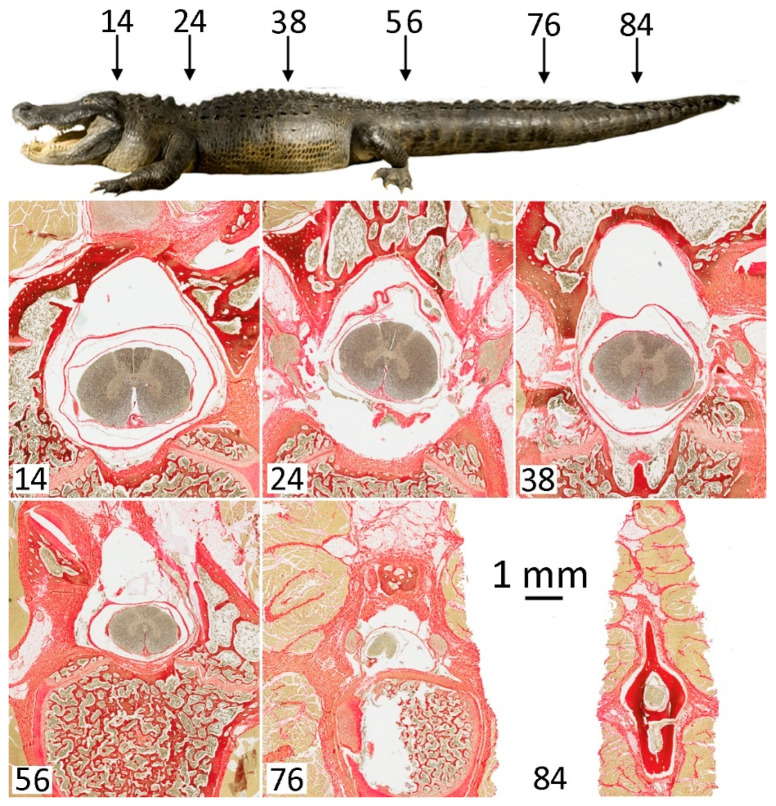
Changes in the size and morphological features along the length of the vertebral column. The position where the sections were taken are illustrated on the figure above and indicated numerically (as % of total body length) in the figures below. Each section is presented at the same magnification, so the scale bar is the same for each figure; note the marked size decrease between the trunk (top row) and caudal/tail sections (bottom row).

In the cervical region, the vertebral canal has an oval shape, due, in part, to the fact that the canal typically reaches its maximum width in this region (Figure 7). The dura is restricted to the ventral portion of the vertebral canal; in transverse sections, the spinal cord has an oblong shape and shows only a slight indication of a cervical enlargement. The transition from the cervical to thoracic region is associated with a decrease in the width of the vertebral canal, as well as the size of the spinal cord and surrounding dura (Figure 7).

In the lumbar region, the vertebral canal increases in size, particularly in the dorsal–ventral plane, being more oblong than oval. The diameter of the dura increases markedly in the lumbar region, as does the diameter of the spinal cord, which exhibits a modest lumbar expansion in the grey matter (Figure 7). From the caudal side of the pelvis to the distal end of the tail, there is a steady decrease in the size of all the measured features (Figure 7). This decrease appears fairly consistent in the vertebral canal and spinal cord, but not so in the dura. The percentage of the vertebral canal filled by dura increases along the tail (Figure 7), and the relationship between dura cross-sectional area and spinal cross-sectional area initially increases before gradually declining.

These trends can be seen in the quantitative summary of the four regions (Table S1). There are two consistent patterns evident in Table S1. As anticipated, the size of the morphological features increased as the alligators increase in size (left to right in Table S1). The second pattern is evident in the individual morphological features, the majority of which showed the same pattern (regardless of the size of the alligator) in having a reduced size in the thoracic region (compared to the cervical or lumbar) and a marked decrease in the mid-caudal region (Table S1).

### 3.2. Trunk

**Figure 8:**
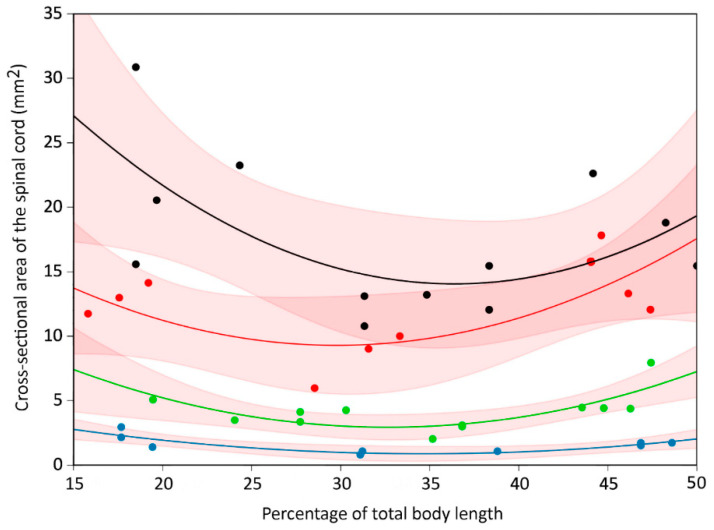
The morphological features in the trunk of *Alligator mississippiensis* were best represented by a parabolic curve. Regardless of size, the features studied were smaller in the thoracic region than in either the cervical or lumbar region. Cross-sectional area of the spinal cord (*Y*-axis) is plotted against % total body length (*X*-axis). Curves are indicated for hatchlings (blue), juveniles (green), sub-adults (red), and adults (black); the 95% confidence interval for each curve is indicated by the pink shading. The hatchling curve only appears flatter because of the scale of the *Y*-axis; at a finer scale, the parabolic curve of each size group is readily apparent.

The second-order polynomial equations used in the trunk region were a compromise intended to reflect the reduced size of the thoracic region (Figure 5) without the addition of excessive variables. The 95% confidence intervals for the polynomial equations from the hatchling and juvenile data sets were consistently discrete; the 95% confidence intervals from the sub-adult and adult data sets typically overlapped, most commonly in the lumbar region (Figure 8). The parameters of the individual polynomial equations for each region and size group are given in Table S2; the consistency among some of the parameters reflects that all of the features were measured from the same sample, and thus shared the same X-value distribution. The relatively narrow range of these parameters (a = 17.23–11.33; b = −0.761–−1.23; c = 0.011–0.020) suggest that a single formula could be used as a reasonable representation of this system, especially for perturbation analysis.

**Figure 9:**
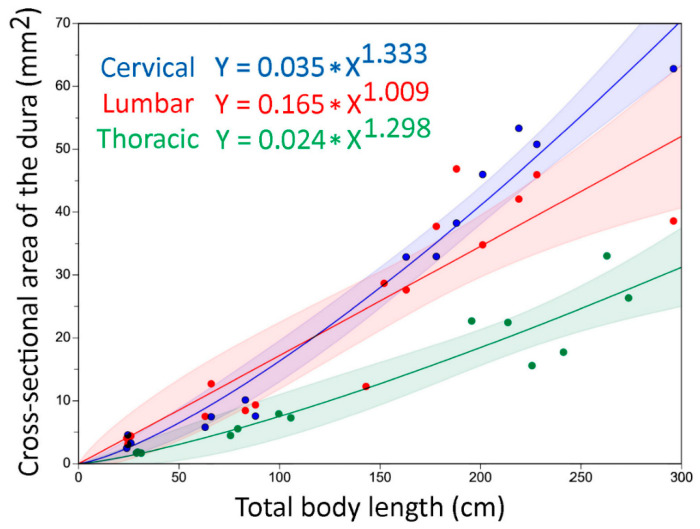
The influence of increasing body size on the morphological features were examined using Power curves (non-linear regression). The cervical and lumbar regions of the dura increase faster than the thoracic dura; this differential growth produces the increasingly prominent regional differences (Figure 8).

Further refinement of the relationship between the morphometric data and the polynomial equations could be gained by incorporating the differential growth rates of the features studied. The parameters of the growth curves (Table S3) indicate that the growth patterns are different in the regions of the trunk. In the cervical and thoracic regions, the growth rates decrease from vertebral canal-dura-spinal cord, indicating that the spinal cord will fill proportionately less of the vertebral canal, and that the subdural space will increase disproportionately in these regions as the animal grows. The relationship between these three growth rates is opposite in the lumbar region (Table S3); this indicates that with increasing animal size the relative size of the lumbar cistern would decrease, as would the “epidural” space. Comparing the growth rates of the same feature in the three different body regions demonstrated the lower absolute growth of the thoracic region (Figure 9), which, ultimately explains the different shapes of the parabolic curves produced by the second-order polynomial equation (Figure 8).

### 3.3. Caudal

As expected, all of the morphological features examined were characterized by a negative linear regression over the length of the tail (Table S4). With the exception of the central canal, there was a regular increase in the value of each parameter with increasing size class of alligator (Table S4). When the samples are restricted to the mid-caudal region, the differential growth rates are clear (Table S5). The vertebral canal, dura, and spinal cord grow at respective lower rates, with the spinal cord being just above isometry (Table S5).

### 3.4. General Trends

**Figure 10:**
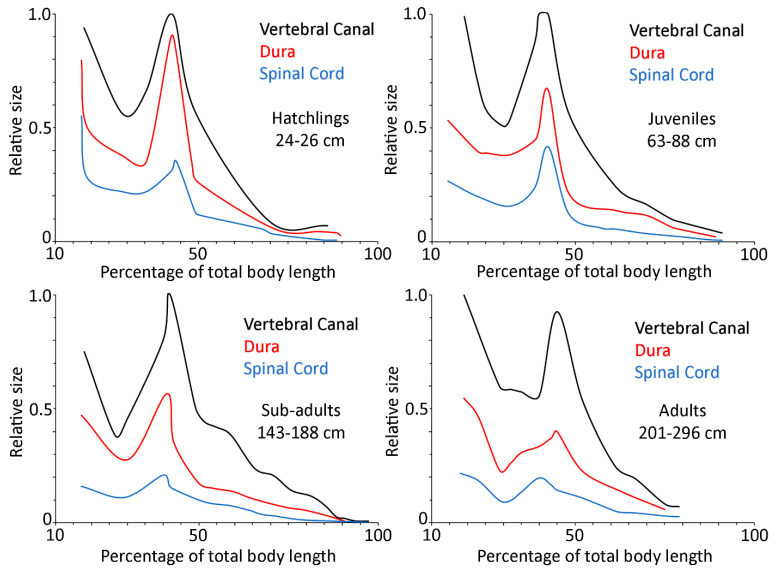
Summary of the regional differentiation and differential growth of the spinal cord and surrounding structures in *Alligator mississippiensis*. The color-coded line for each structure (vertebral canal, black; dura, red; spinal cord, blue) is not horizontal, indicating the regional differentiation. The lines are different in each of the four size classes, reflecting the differential growth patterns. Within each size class the morphological features are depicted as relative to the maximum size of any feature from that class.

To standardize a discussion of general trends, the main features were presented as relative size against percent body length for the four size classes of alligators (Figure 10). One of the most remarkable trends is the relative decrease in the area of the spinal cord; in the hatchlings, the cervical and lumbar expansions are evident, while in the adult, there is more of a thoracic restriction than a distinct expansion. The lumbar dura also goes through a significant decrease in relative size as the alligators grow. The lumbar cistern can be defined as the subdural space between the lumbar dura and lumbar spinal cord lines in Figure 10; the relative volume of this cistern decreases with increasing size of the alligator. The relative position of the pelvis shifts slightly as the alligator grows, which displaces the lumbar region relatively caudally (Figure 10). The caudal third of the tail of hatchling alligators is relatively straight, compared to the more tapered tail seen in the other size classes; accordingly, there is a steeper slope between the lumbar peak and the mid-caudal region in the hatchling than in the larger size classes (Figure 10).

**Figure 11:**
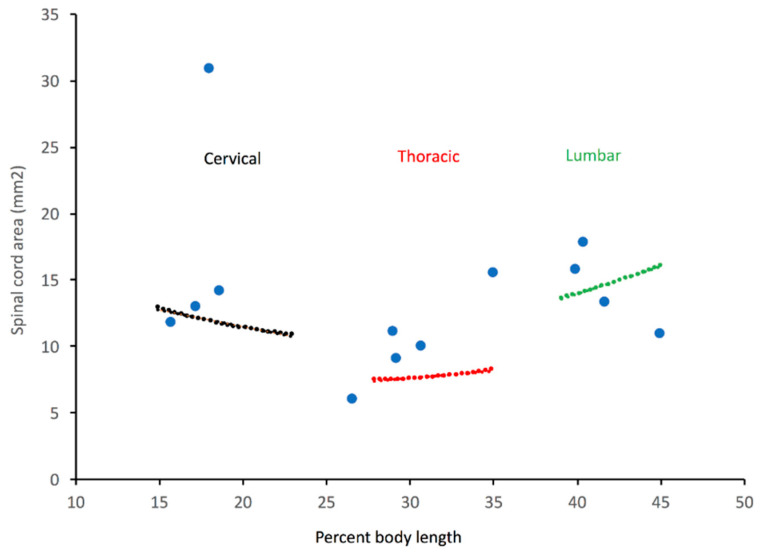
Predicted and measured sizes of the spinal cord of *Alligator mississippiensis*. The colored dots and lines represent predicted values based on the polynomial curve from juvenile specimens scaled up to the mean size of the sub-adult specimens using the calculated regression coefficients from the three regions. Measured values from the sub-adult specimens are indicated by blue circles.

The morphometric data set assembled for *Alligator mississippiensis* was designed to be scalable by region or structure. For example, the polynomial curve for spinal cord area in juvenile specimens (Table S2) can be used as a starting point, then the values scaled, using the allometric parameters specific for the spinal cord in the three regions of the trunk (Table S3), up 101 cm of body size to reach the mean of the sub-adult specimens. The resulting predictions for spinal cord area demonstrate a good fit with the measured data (Figure 11).

### 3.5. Impact of Histological Methodology

**Figure 12:**
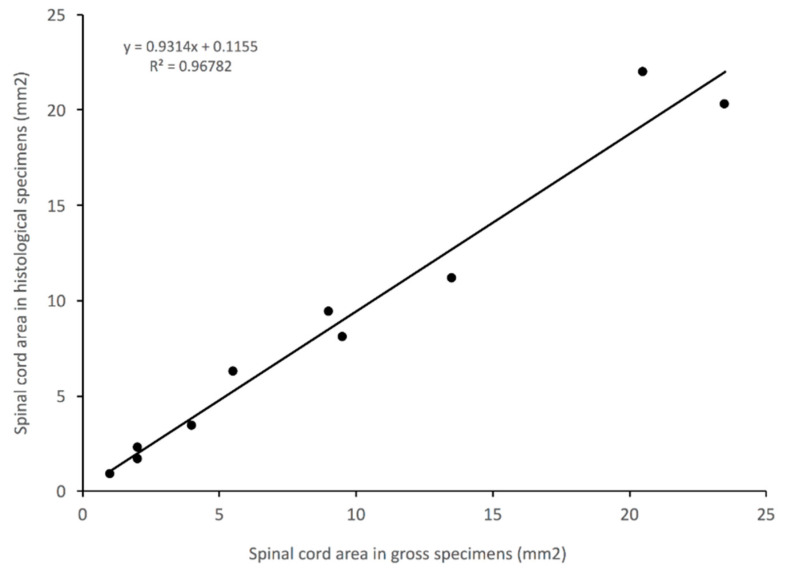
Distortion of the morphology produced by histological sectioning. The same sections of sub-adult spinal cord cross-section were measured first as gross sections (*X*-axis), and then as histological sections (*Y*-axis). The deviation of the slope of the curve away from 1 indicates a distortion of roughly 7% associated with the histological processing.

Selected sections were photographed fresh, then again after histological sectioning and staining (as detailed above). The quantification of both digital images (Figure 12) indicates a shrinkage of approximately 7% during the histological processing.

## 4. Discussion

This study was undertaken to document changes in the dimensions of the vertebral canal, spinal cord, and dura both along the axial length of *Alligator mississippiensis*, and as the alligator increased in size. All of the structural features quantified had a similar pattern along the axial length of the alligator. The cervical and lumbar regions were the largest (and were typically similar in size), while the thoracic region was significantly smaller; the proximal end of the caudal region was similar to the thoracic region in size, but the features all decreased over the length of the tail. By comparing the same structural feature across a large size range of alligators, the present study was able to document differential growth rates. These differential growth rates both maintained the different regions (e.g., the thoracic spinal cord grew at a lower rate than the cervical spinal cord), but also created distinct differences, particularly in the relative reduction of the lumbar cistern with increasing size of the alligator (Figure 10).

**Figure 13:**
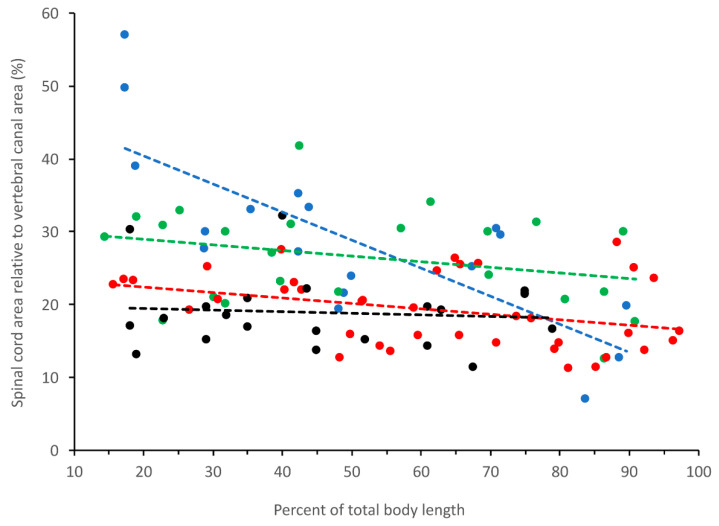
Spinal index, the cross-sectional area of the spinal cord as a percent of the cross-sectional area of the vertebral canal. Data for each size class (and a linear best-fit line) are indicated by color (hatchlings, blue; juveniles, green; sub-adults, red; adults, black). In the hatchlings the percentage size of the spinal cord decreases over the length of the body, while in the other three size classes the percentage remains relatively stable along the length of the body. The differential scaling of the vertebral canal and spinal cord results in each size class of specimens having slightly different percentage relationship between these two morphological features.

The earlier morphometric study of the alligator spinal cord [28] described a clear distinction between cervical, thoracic, and lumbar regions. One goal of that earlier study was to establish an index value for the relationship between spinal and vertebral areas; Giffin reported that the spinal cord of *Alligator* filled between 47 and 38% of the vertebral canal [28]. The present study examined more of the axial length of the spinal cord (as it included the caudal region), and included specimens from a larger range of sizes. The present study found that in hatchling alligators, the cervical spinal cord can exceed 51% of the vertebral canal, but this decreased to 15% over the length of the animal (Figure 13). The percentage of the vertebral canal filled by the spinal cord decreases with increasing body size of the alligator (as indicated by the differential scaling values), and tended to be more consistent over the length of the alligator’s vertebral canal (Figure 13). The present study found that area of the vertebral canal increased with body size in the cervical, thoracic, and mid-caudal regions, but decreased relative to body size in the lumbar region; this lumbar decrease has been found in other crocodilians [42].

The majority of the vertebral canal of *Alligator mississippiensis* is occupied by a spinal venous complex [43] and the dural sheath of the spinal cord (Figure 3). The relative size of the dura was not consistent along the length of the spinal cord; the lumbar expansion of the dura represents a localized increase in the subdural space, typically referred to as a cistern [44]. A “lumbar” cistern is a common feature of the mammalian and avian CNS [45], but the mammalian and avian cisterns are relatively short, blind ending structures (though the cistern is associated with a special balance apparatus in avians [46]). In *Alligator mississippiensis* the lumbar cistern is not blind ending, being continuous with the caudal subdural space, which extends for roughly half the body length of the animal.

**Figure 14:**
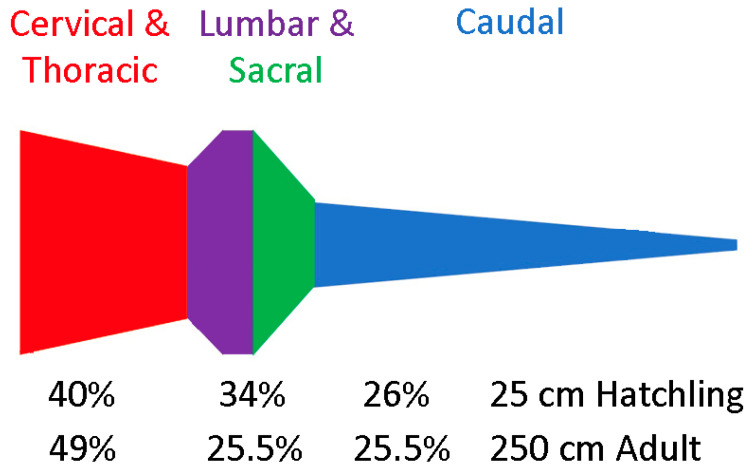
The marked decrease in the lumbar cistern with increasing size in *Alligator mississippiensis*. The size of the subdural space is depicted along the length of the vertebral column; note the stability of the relative size of the subdural space in the tail. In this figure, and the underlying calculations, the sacral region was defined as the percentage of body length between the caudal margin of the lumbar region and the cranial margin of the caudal series, and was modeled as a trapezoid between the lumbar and caudal sizes.

The relative size of the lumbar cistern shifts with increasing body size of *Alligator mississippiensis*. If the difference between the dura and spinal cord areas is taken, it yields the area of the subdural space (Figure 14). It is important to note that these represent potential volumes, not the actual volume of CSF. Still, Figure 14 clearly reflects the large size of the lumbar cistern in the hatchling; the combined lumbar and sacral regions make up only 13% of the body length, but have a subdural volume nearly equal to the cervical and thoracic regions which make up 23% of the body. The relative change in size of the lumbar cistern with growth is also evident in Figure 14; note that the decrease in the volume of the lumbar cistern (from 34 to 25.5%) is due primarily to a nearly isometric growth pattern in the lumbar dura combined with a slight positive allometry of the lumbar spinal cord. This means that unlike the cervical and thoracic regions, where dural growth exceeds spinal growth, in the lumbar region the subdural space exhibits negative allometry.

Though beyond the scope of the present contribution, the structure and size of the lumbar cistern in *Alligator mississippiensis* raise fascinating questions about the functional role of such a cistern and the associated spinal CSF circulatory mechanics. In mammals, the lumbar cistern can function as a “relief valve” for the cranial CSF; increasing CSF pressure causes a shift of CSF from the cranial subarachnoid and an expansion of the lumbar cistern [47]. It is not clear how a cistern that is not blind ending could function as a relief valve.

The underlying mechanics remain to be fully established, but there is solid experimental evidence that in mammals there is CSF flow in the spinal subarachnoid spaces which is linked to the ventilator cycle [9]. It is not clear how the mechanics associated with spinal CSF flow in the trunk could cross the lumbar cistern. Similarly, the mechanics for CSF flow along the caudal spinal cord, which extends for nearly half the length of the animal, have never been explored.

The regression values for the spinal cord area along the length of the tail (Table S4) reveal increasing negative allometry with increasing body size. The magnitude of the negative allometry is such that between 50% of the body length and 68% of the body length (the middle of the mid-caudal region) the spinal cord area decreases by 50% in hatchlings and by over 55% in adult alligators. The tail base in crocodilians supports expanded axial muscles capable of oscillating the heavy tail [48]. As *Alligator mississippiensis* increases in body size, they are increasingly aquatic [49] and increasingly rely solely on their tails for aquatic propulsion [50]. The spinal cord area at the tail base has a greater regression coefficient (1.200) than at the mid-caudal level (Table S5), suggesting that there is a modest caudal expansion to the alligator spinal cord (near the 28th and 29th vertebrae, Figure 4), which is directly correlated with swimming capacity. Wedel et al. [51] proposed a similar hypothesis for the caudal spine cord of *Haplocanthosaurus*, but in that case there is no obvious ecological or locomotor explanation for increased use of the basal tail musculature.

This study relied on histological analysis. Comparison of gross and histological sections (Figure 12) indicates a 7% distortion associated with histological processing. This value is comparable to previously published levels [37], and can be accommodated within any typical perturbation analysis. One potential cost of our histological approach was a solid data set on the morphometrics of the central canal of the spinal cord. Our data on the diameter of the central canal had a greater level of variation than any other data set, including multiple sections in which no central canal could be discerned (Table S1). Similar levels of morphological variation (including the absence of a central canal) have been described in humans and other vertebrates [52,53].

One of the rationales for this study was to construct a morphometric data set that could be used for computational fluid dynamics modeling of the *Alligator* spinal cord. A common approach to this modeling [54,55] is Poiseuille’s law:

CSF velocity = ΔCSF pressure/{4μ [(R_1_^2^ − r^2^) + (R_2_^2^ − R_1_^2^) ∗ (log[r/R_1_]/log[R_2_/R_1_])]}

In which μ (the dynamic viscosity) is a constant, R_1_ is the radius of the spinal cord, R_2_ is the radius of the dura, and r is the radius to the center of the subdural space. The radi values for the spinal cord, dura, and mid-subdural can be modeled along the length of the spinal cord using the mean values and polynomial curves (Tables S1, S2 and S4) and the influence of allometric changes can be modeled by incorporating the differential allometric growth coefficients (Tables S3 and S5).

## 5. Conclusions

All of the structural features quantified had a similar pattern along the axial length of *Alligator mississippiensis*. The cervical and lumbar regions were the largest (and were typically similar in size), while the thoracic region was significantly smaller; the proximal end of the caudal region was similar to the thoracic region in size, but the features all decreased over the length of the tail. By comparing the same structural feature across a large size range of alligators, the present study was able to document differential growth rates. These differential growth rates both maintained the different regions (e.g., the thoracic spinal cord grew at a lower rate than the cervical spinal cord), but also created distinct difference, particularly in the relative reduction of the lumbar cistern with increasing size of the alligator. This morphometric data set both documents the different sizes and growths within the spinal system of *Alligator*, and forms a quantitative foundation for biophysical modeling of this system.

## Data Availability

The data are available through reasonable request to the corresponding author.

## Supplementary Materials

The following supporting information can be downloaded at: https://www.mdpi.com/article/10.3390/biology11040514/s1, Table S1: Summary of the morphometric data from *Alligator mississippiensis*. The morphometric data is presented as mean ± standard error. For vertebral canal, dura, spinal cord area, and subdural space the data are given as mm^2^; for central canal area, the data are given as μm^2^. The results of the ANOVA are presented as F value, degrees of freedom. The calculated *p* value is for the hypothesis that there is no differences among the morphometric data by sampling location. The results of the post hoc Bonferroni test are listed with only the comparisons found to be insignificant listed. The MANOVA comparisons that were found to be not significant are indicated by blanks, note that these are restricted to the area of the central canal; Table S2: Summary of the second-order polynomial curves used to fit the morphometric data from the trunk region. The three variables associated with the shape and size of the parabolic curve (a,b,c) are listed, as are the three main metrics for judging the relative fit of the curve: the standard error (s.e.), the correlation coefficient (R), and the Akaike Information coefficient (AICC); Table S3: Summary of the Power curves used to scale the morphometric data across the size range of *Alligator mississippiensis* specimens. The two variables associated with the shape and size of the curve (a,b) are listed, as are the three main metrics for judging the relative fit of the curve: the standard error (s.e.), the correlation coefficient (R), and the Akaike Information coefficient (AICC); Table S4: Summary of the linear regression curves used to scale the morphometric data along the caudal series/tail length of *Alligator mississippiensis*. The two variables associated with the shape and size of the curve (a,b) are listed, as are the three main metrics for judging the relative fit of the curve: the standard error (s.e.), the correlation coefficient (R), and the Akaike Information coefficient (AICC); Table S5: Summary of the Power curves used to scale the caudal morphometric data across the size range of *Alligator mississippiensis* specimens. The two variables associated with the shape and size of the curve (a,b) are listed, as are the three main metrics for judging the relative fit of the curve: the standard error (s.e.), the correlation coefficient (R), and the Akaike Information coefficient (AICC).
